# Autophagy regulates the survival of cells with chromosomal instability

**DOI:** 10.18632/oncotarget.11736

**Published:** 2016-08-31

**Authors:** Dawei Liu, Zeeshan Shaukat, Tianqi Xu, Donna Denton, Robert Saint, Stephen Gregory

**Affiliations:** ^1^ Department of Genetics, University of Adelaide, Adelaide, SA, Australia; ^2^ Centre for Cancer Biology, University of South Australia, Adelaide, SA, Australia; ^3^ Flinders University, Adelaide, SA, Australia

**Keywords:** chromosomal instability, autophagy, mitophagy, parkin, drosophila

## Abstract

Chromosomal instability (CIN) refers to genomic instability in which cells have gained or lost chromosomes or chromosomal fragments. A high level of CIN is common in solid tumours and is associated with cancer drug resistance and poor prognosis. The impact of CIN-induced stress and the resulting cellular responses are only just beginning to emerge. Using proliferating tissue in *Drosophila* as a model, we found that autophagy is activated in CIN cells and is necessary for their survival. Specifically, increasing the removal of defective mitochondria by mitophagy is able to lower levels of reactive oxygen species and the resultant cellular damage that is normally seen in CIN cells. In response to DNA damage, CIN is increased in a positive feedback loop, and we found that increasing autophagy by Tor depletion could decrease the level of CIN in proliferating cells. These findings underline the importance of autophagy control in the development of CIN tumours.

## INTRODUCTION

Chromosomal instability (CIN) refers to the process by which cells are unable to maintain chromosomal integrity or number [1]. Chromosomal instability (CIN) or genomic instability (GIN) has been suggested as a pivot hallmark of cancer which facilitates the acquisition of other cancer hallmarks required for tumorigenesis [2]. CIN is seen in most human solid tumours and the genetic variation it generates can account for the development of drug resistance and the poor prognosis of CIN cancer patients [3, 4]. It has been proposed that CIN itself could be an attractive target for chemotherapy, as it is a relatively cancer-specific phenotype [5–7]. However, little is known about which properties of CIN cells might allow CIN tumours to be efficiently killed.

Autophagy is a normal cellular pathway for the degradation and recycling of unnecessary or dysfunctional cellular components [8–10]. The process of autophagy involves the sequestration of cytoplasmic material by double-membrane phagophores to form autophagosomes that then fuse with lysosomes to enable degradation of their cargo [8]. Autophagy is induced in response to various stresses to maintain metabolic homeostasis and prevent the build-up of dysfunctional cellular components [9]. The aberrant regulation of autophagy has been seen in several diseases, especially in neurodegenerative disease and cancer [10–12], as well as in cells in which aneuploidy has been induced [13–15]. However, whether autophagy is protective or deleterious in the development of cancer has been widely debated [16]. The information currently available from clinical trials and mouse models suggests that a lack of autophagy predisposes tissue to develop tumours, possibly because autophagy normally moderates oxidative stress and DNA damage by removing defective mitochondria. However, in some model systems, autophagy is essential for the growth of the tumour [17, 18]. Consequently there are now ongoing clinical trials evaluating the combination of inhibition of autophagy with chemotherapeutics [19, 20]. The expectation is that tumours may need autophagy to tolerate the metabolic demands of proliferation, to avoid excessive oxidative stress and consequently an unmanageable level of genome instability. Thus reduced autophagy may promote tumorigenesis by increasing DNA damage rates, but for tumours to thrive they may need to increase their autophagic flux to prevent deleterious levels of oxidative damage.

In characterizing pathways which facilitate the survival of CIN cells, we have previously reported that CIN cells are sensitive to changes in glycolysis or antioxidant enzymes and generate elevated levels of reactive oxygen species [21]. Based on that study, we carried out further screening for candidates whose depletion can specifically kill CIN cells. In this process, we found that when CIN is induced in otherwise normal cells, they become sensitive to the depletion of autophagy. Here we show that CIN leads to an increase in autophagy, and that autophagy is needed to limit reactive oxygen species, DNA damage and cell death in CIN cells. Furthermore, elevated levels of autophagy promote the survival of CIN cells.

Altogether, our research highlights the significance of understanding autophagy pathways as a potential therapeutic target for the treatment of CIN tumours.

## RESULTS

### Autophagy is activated when CIN is induced in proliferating cells

**Figure 1:**
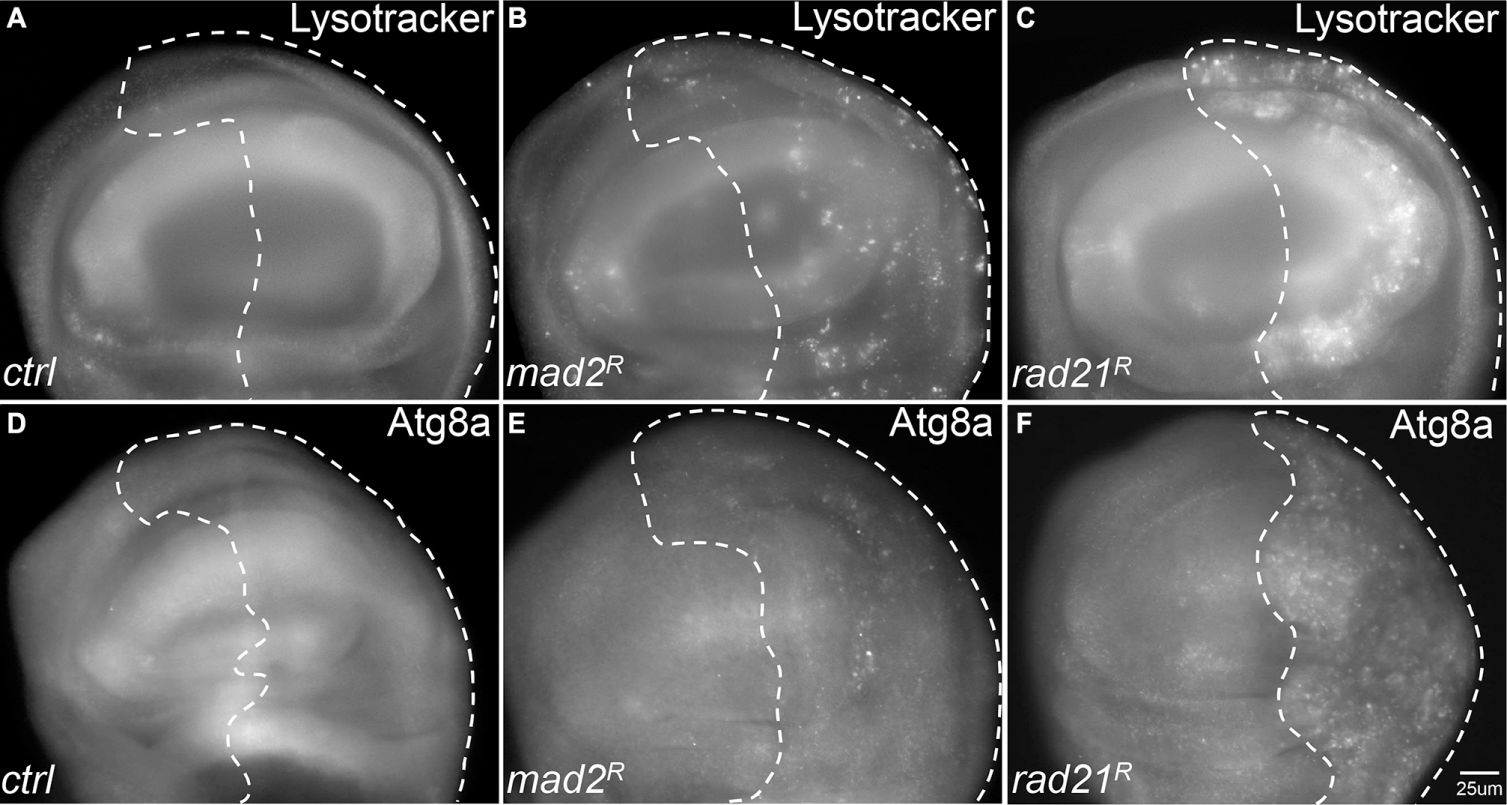
Autophagy is activated in tissues with Chromosomal Instability (CIN) CIN was induced in the posterior half of each wing disc as indicated by the dotted line (marked by the expression of *UAS-CD8-GFP*) while the rest of each disc was wild type. (**A**–**C**) Lysotracker staining of third instar larval wing discs. Wing discs with CIN induced by either Mad2 depletion (B, *engrailed* > *Gal4*, *UAS-CD8-GFP, UAS-mad2^RNAi^*) or Rad21 depletion (C, *engrailed* > *Gal4*, *UAS-CD8-GFP, UAS-rad21^RNAi^ UAS-Dicer2*) showed increased lysosome staining relative to the control (A, *engrailed* > *Gal4*, *UAS-CD8-GFP*). Representative discs are shown; the phenotype was observed all Mad2 depleted discs tested (11) and all Rad21 depleted discs (21) but no control discs (0 from 7). (**D**–**F**) The level of mCherry-Atg8a in third instar larval wing discs. Wing discs with CIN induced by either Mad2 depletion (e, *engrailed* > *Gal4*, *UAS-CD8-GFP, UAS-mCherry-Atg8 UAS-mad2^RNAi^*) or Rad21 depletion (F, *engrailed* > *Gal4*, *UAS-CD8-GFP, UAS-mCherry-Atg8*, *UAS-rad21^RNAi^ UAS-Dicer2*) showed increased induction of autophagy relative to the control (D, *engrailed* > *Gal4*, *UAS-CD8-GFP*) as indicated by the level of mCherry-Atg8a puncta. This phenotype of differing puncta from the wild type half was observed in all Mad2 depleted discs tested (5) and all Rad21 depleted discs (39) but no control discs (0 from 8).

We have previously used RNA interference knockdown of the spindle assembly checkpoint gene *mad2* or cohesin gene *rad21* to generate inducible CIN models with different CIN levels [22]. From this work, and that of others [23] it has become clear that aneuploidy is associated with elevated levels of reactive oxygen species (ROS). We expected that in response, CIN cells would induce autophagy to recycle damaged macromolecules. To test autophagy levels in cells with induced CIN, we initially used lysotracker staining, which was elevated in both *mad2* and *rad21* CIN cells relative to normally proliferating cells (Figure 1A–1C). To confirm this result we examined the levels of a tagged form of Atg8a [24]. In line with the lysotracker staining, we found robust Atg8a puncta formation in *rad21* CIN cells indicating autophagy activation (Figure 1F). Stronger induction of autophagy was seen in *rad21* CIN cells than in *mad2* CIN cells (Figure 1A–1F), consistent with the higher level of CIN generated in the *rad21* model [22].

### Reducing autophagy leads to increased oxidative stress and apoptosis in CIN cells

**Figure 2:**
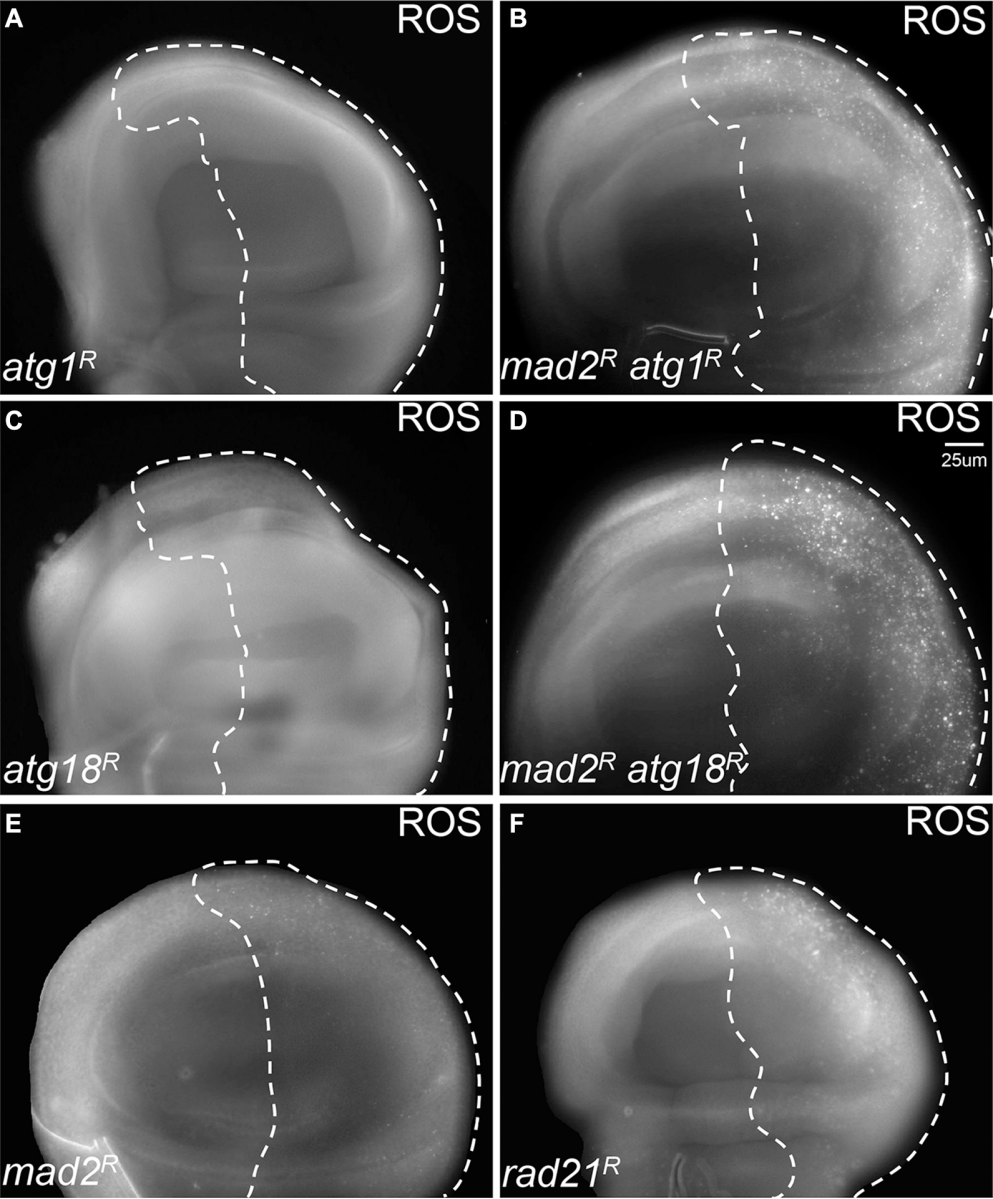
Blocking autophagy causes redox stress in CIN cells CellRox staining was used to detect the level of oxidative stress. The indicated genes were knocked down in the posterior half of each wing disc as indicated by the dotted line while the rest of each disc was wild type. Knocking down either Atg1 ((**A**) *engrailed* > *Gal4*, *UAS-CD8-GFP,* UAS-*atg1^RNAi^*) or Atg18 ((**C**) *engrailed* > *Gal4*, *UAS-CD8-GFP,* UAS-*atg18^RNAi^*) did not give oxidative stress, and the CellRox signal was low or absent in *mad2^RNAi^* CIN cells ((**E**) *engrailed* > *Gal4*, *UAS-CD8-GFP, UAS-mad2^RNAi^*). However, when Atg1 ((**B**), *engrailed* > *Gal4*, *UAS-CD8-GFP, UAS-mad2^RNAi^*, UAS-*atg1^RNAi^*) or Atg18 ((**D**), *engrailed* > *Gal4*, *UAS-CD8-GFP, UAS-mad2^RNAi^*, UAS-*atg18^RNAi^*) were depleted in CIN cells, an elevated level of oxidative stress was observed. Depletion of *rad21* (**F**, *engrailed > Gal4*, *UAS-CD8-GFP*, UAS-*rad21^RNAi^*, UAS-*Dicer2*) shows for comparison the elevated ROS generated by a high CIN rate. Representative discs are shown; a clear difference from the wild type anterior half was observed in all discs when Atg1 (11 discs tested) or Atg18 (7 tested) were depleted with Mad2, but none of the control discs (0 from 10 for Atg1 alone; 0 from 9 for Atg18 alone; 0 from 13 for Mad2 alone).

**Figure 3:**
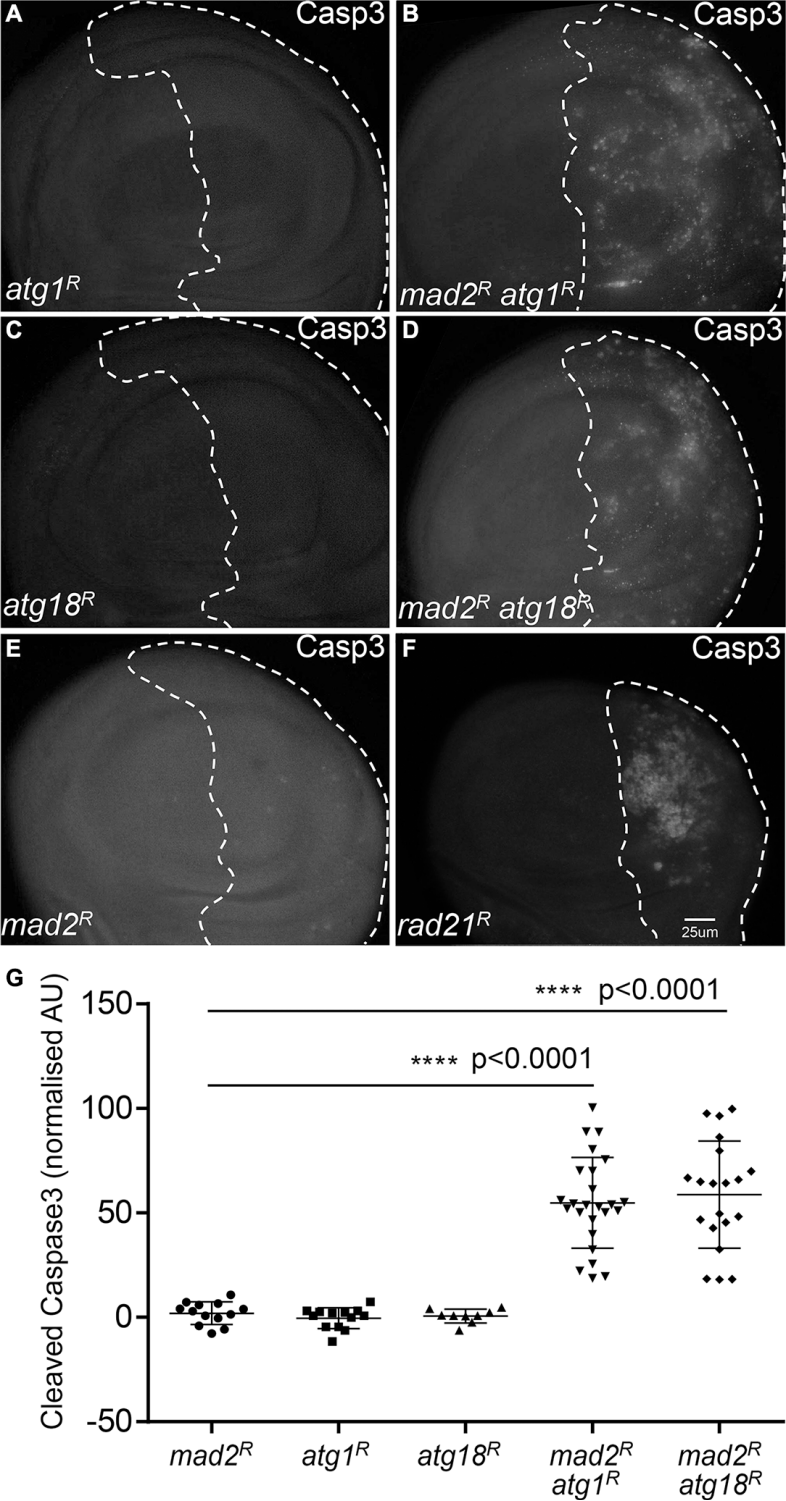
Blocking autophagy increases cell death in CIN cells Anti-cleaved caspase3 antibody staining was used to show the level of apoptosis. The indicated genes were knocked down in the posterior half of each wing disc as indicated by the dotted line and the rest of each disc was wild type. Knocking down either Atg1 ((**A**) *engrailed* > *Gal4*, *UAS-CD8-GFP,* UAS-*Atg1^RNAi^*) or Atg18 ((**C**) *engrailed* > *Gal4*, *UAS-CD8-GFP,* UAS-*Atg18^RNAi^*) did not cause apoptosis in these proliferating cells. However, knocking down Atg1 ((**B**) *engrailed* > *Gal4*, *UAS-CD8-GFP, UAS-mad2^RNAi^*, UAS-*atg1RNAi*) or Atg18 ((**D**) *engrailed* > *Gal4*, *UAS-CD8-GFP, UAS-mad2^RNAi^*, UAS-*Atg18^RNAi^*) in CIN cells, significantly increased the level of apoptosis in these cells relative to the CIN alone control (B, *engrailed* > *Gal4*, *UAS-CD8-GFP, UAS-mad2^RNAi^*). Depletion of *rad21* ((**F**) *engrailed > Gal4*, *UAS-CD8-GFP*, UAS-*rad21^RNAi^*, UAS-*Dicer2*) shows for comparison the elevated apoptosis generated by a high CIN rate. Quantification of the cleaved caspase3 staining is shown in (**G**). In all cases *n* ≥ 9 and the error bars show 95% confidence intervals around the mean. The *p* values were calculated using two-tailed *t*-tests with Welch's correction.

Having found that autophagy is activated in CIN cells, we hypothesized that robust autophagy activation might be particularly needed for the survival of CIN cells. In order to address this hypothesis, we depleted the essential autophagy genes *Atg1* or *Atg18a* [25, 26] by RNA interference in CIN cells. Atg1 is needed for a functional autophagy induction complex and leads to the recruitment of Atg18/WIPI2, which is needed for Atg8 recruitment and phagosome function [25, 27]. We found that knocking down either *Atg1* or *Atg18* led to dramatically increased levels of oxidative stress and DNA damage in CIN cells (Figure 2, Supplementary Figure S1). Furthermore, depletion of *Atg1* or *Atg18* in CIN cells resulted in a significant increase in apoptosis as detected by active caspase staining (Figure 3). Elevated levels of cell death were seen when autophagy was blocked in either CIN model (Supplementary Figure S2). However, depleting *Atg1* or *Atg18* in normal proliferating cells had no detectable effect on ROS levels, DNA damage or apoptosis. These results are consistent with a protective role for autophagy in response to cellular stresses [28], and showed that that autophagy activation was required for the survival of CIN cells.

### Enhancing autophagic flux rescues oxidative stress levels and apoptosis in CIN cells

**Figure 4:**
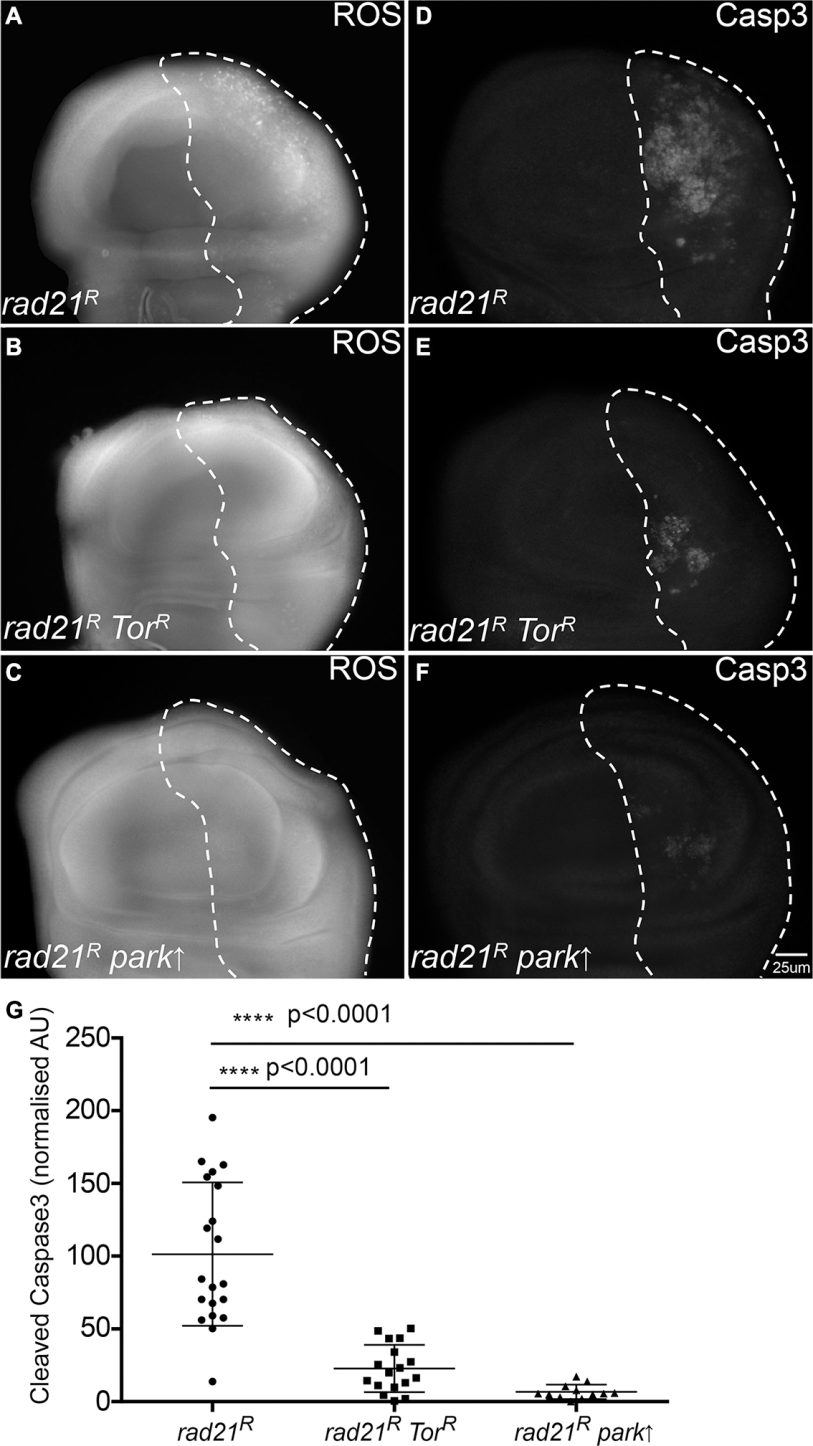
Enhancing autophagy or mitophagy decreases redox stress and cell death in CIN cells Anti-cleaved caspase3 antibody was used to stain the level of apoptosis in cells while CellRox staining was used to detect the level of oxidative stress. The indicated genes were knocked down in the posterior half of each wing disc as indicated by the dotted line and the rest of each disc was wild type. Enhancing autophagy by Tor knockdown (*engrailed > Gal4*, *UAS-CD8-GFP,* UAS-*Tor^RNAi^*, UAS-*rad21^RNAi^*, UAS-*Dicer2*) (**B**, **E**) reduced the level of oxidative stress (B) and apoptosis (E) observed in CIN cells relative to the CIN alone controls (**A**, **D**). A similar reduction in oxidative stress (**C**) and apoptosis (**F**) was observed in CIN cells when mitophagy was induced by the overexpression of Parkin (*engrailed > Gal4*, *UAS-CD8-GFP,* UAS-*parkin*, UAS-*rad21^RNAi^*, UAS-*Dicer2*). Quantification of the cleaved caspase3 staining is shown in (**G**). In all cases *n* ≥ 12 and the error bars show 95% confidence intervals around the mean. The *p* values were calculated using two-tailed *t*-tests with Welch's correction.

Having observed that CIN cells required autophagy to avoid cell death, we wished to see whether enhancing autophagic flux could improve the survival of CIN cells. Autophagy induction is regulated by conserved upstream signalling pathways that converge on the target of rapamycin (TOR) kinase, which prevents autophagy by inhibiting Atg1 [26, 29]. By the removal of the autophagy inhibitor Tor using RNAi, we found that enhancing autophagic flux (Supplementary Figure S4) could rescue the oxidative stress and apoptosis phenotype in CIN cells (Figure 4, Supplementary Figure S3). This suggested that autophagy is not normally induced enough to protect cells with high levels of CIN, and that elevated autophagy, which is often seen in cancer [7, 8], can improve the survival of these cells.

### Autophagy of mitochondria is needed in CIN cells to prevent ROS and cell death

**Figure 5:**
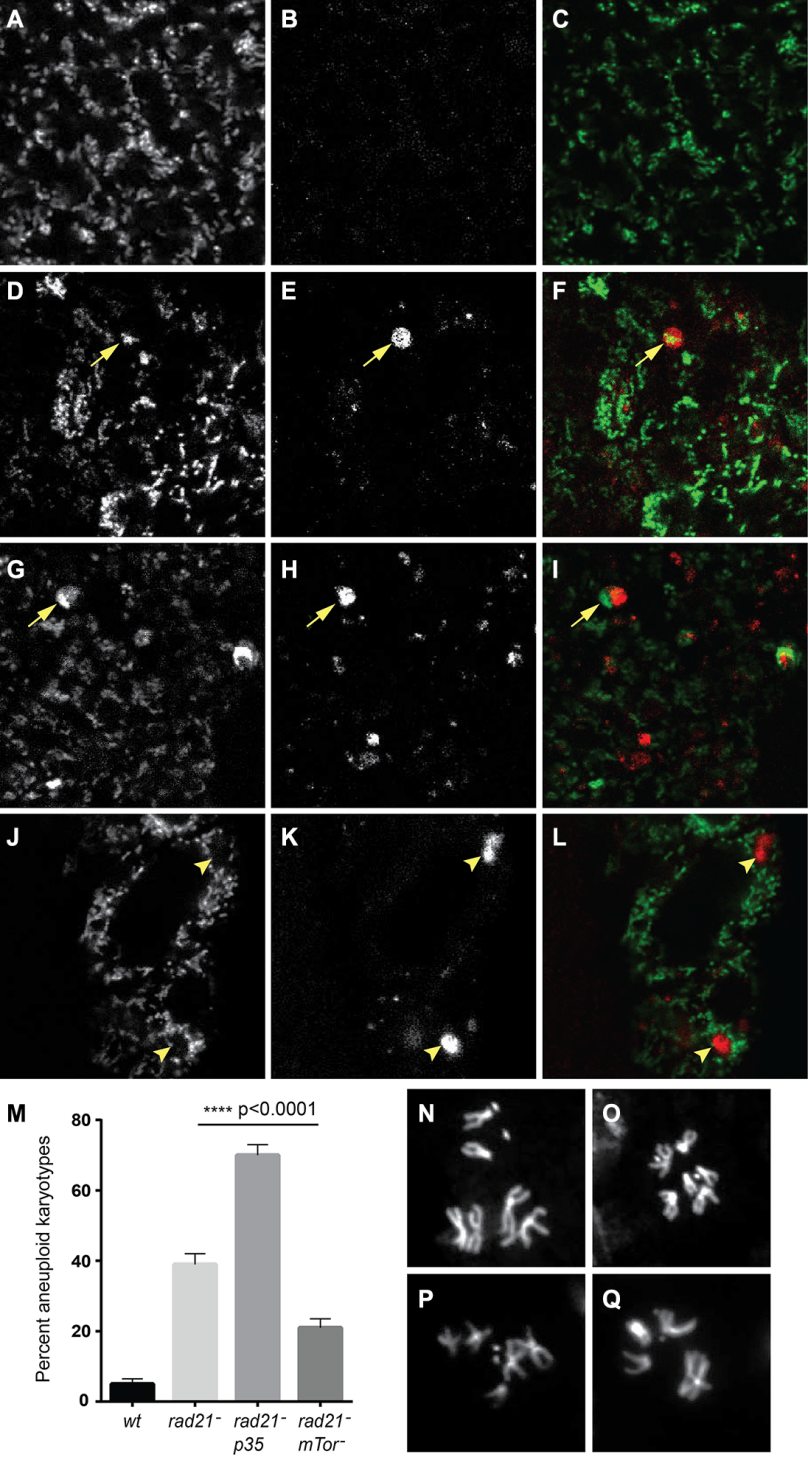
Autophagy of mitochondria is observed in CIN cells, and increased autophagy decreases CIN levels Mito-GFP (**A**, **D**, **G**, **J**) was used to mark mitochondria and mCherry-Atg8 (**B**, **E**, **H**, **K**) was used to detect autophagosomes in third instar larval wing discs. Merged images are shown in (**C**, **F**, **I**, **L**) with mito-GFP in green and mCherry-Atg8 in red. CIN cells induced by Rad21 depletion (D–I, *btub > mito-GFP*, UAS-*mCherry-Atg8*, *engrailed > Gal4*, UAS-*rad21^RNAi^*, UAS-*Dicer2*) showed co-localization (arrowed) of mitochondria (F) and large autophagosomes (G) while such autophagosomes were not seen in cells without CIN (A–C, *btub > mito-GFP*, UAS-*mCherry-Atg8*, *engrailed > Gal4*). Some cells showed interruption of the mitochondrial network by autophagosomes (J–L, arrowheads), or decreased mito-GFP signal from mitochondria in the area containing an autophagosome (G–I, arrowed). The level of CIN was evaluated by the frequency of aneuploid metaphases (**M**). Rad21 depletion gave aneuploidy in 39% of metaphase cells, or 70% if cell death was blocked by expression of p35, while the level of CIN in Rad21 depleted cells could be significantly reduced to 22% by Tor knock down. The *p value* was calculated using Fisher's exact test, *n* > 240 for each genotype. Representative control euploid (**N**, **O**) and CIN cell aneuploid karyotypes (**P**, **Q**) are shown.

One function of autophagy activation is the removal of defective mitochondria through *pink1*/*parkin*-mediated mitophagy [30]. CIN is known to cause defective mitochondria and increased oxidative stress in cells [21, 22], therefore, we checked whether mitophagy is involved in the response to CIN. We found that overexpression of the essential mitophagy gene *parkin* reduced the level of ROS and apoptosis in CIN cells at least as effectively as increasing general autophagy by *Tor* depletion (Figure 4). Consistent with this, depletion of Parkin significantly increased apoptosis in CIN cells, but not normal cells (Supplementary Figure S5). If removal of defective mitochondria is an essential function in CIN cells, we would expect to detect mitochondria being processed by autophagy in CIN cells. To test this we visualized autophagosomes with mCherry-Atg8 and mitochondria with mito-GFP (Figure 5). In CIN cells we observed cytoplasmic accumulations of Atg8, marking the autophagosomes, and in approximately 20% of cases (127 of 600) they contained mito-GFP. In some cells the mitochondrial network appeared to be interrupted by the presence of the autophagosomes, or the mito-GFP signal was compromised where it fell in an autophagosome relative to the adjacent mitochondria. Control cells did not have large autophagosomes or any striking co-localization with mito-GFP. These results suggest that mitochondria in CIN cells can be removed by autophagy and that this process is necessary for the survival of CIN cells.

### Enhancing autophagic flux reduces the level of CIN in proliferating cells

It has been reported that defective autophagy increases the level of CIN in cancer cells due to increased DNA damage and gene amplification [31]. Conversely, we would expect treatments that decrease DNA damage to lower CIN levels. As enhancing autophagic flux reduced the level of ROS (Figure 4), and we have previously shown that DNA damage in CIN cells is caused by elevated ROS [21], we wished to test whether increasing autophagy could moderate the CIN level. In order to address this hypothesis, we checked the frequency of aneuploid metaphases after autophagy enhancement and compared them with the frequency seen in CIN cells or CIN cells in which cell death had been blocked by expressing the apoptosis inhibitor p35. Blocking apoptosis allowed the retention of many more aneuploid cells in CIN tissue, but by contrast we found that enhanced autophagic flux could significantly reduce the CIN level in a proliferating tissue (Figure 5).

## DISCUSSION

Autophagy can function as a pro-survival protective pathway in cancer cells to fulfil their metabolic demands for rapid cell proliferation and to respond to cellular stresses that may include genomic instability and metabolic stress [31–34]. Therefore, we assessed the level of autophagy in cells with induced CIN and found a robust activation of autophagy (Figure 1). As would be expected for a tissue with random mitotic defects, not every cell showed elevated autophagy. The frequency of elevated autophagy was significantly higher in the high-CIN cells generated by Rad21 depletion, which also have a high frequency of elevated ROS generation, DNA damage and aneuploidy [22]. This is consistent with data from human cells showing that increased levels of aneuploidy correlate with elevated Atg8/LC3 and p62 [13, 14]. In that work, the effect of ongoing karyotypic variation (CIN) on autophagy was not tested, possibly because it is difficult to maintain proliferation in vertebrate CIN cells without additional changes such as p53 loss [1], which would itself impact autophagy. In the case of CIN cells, we found that p62 levels are decreased, indicating effective clearance by higher autophagic flux, as opposed to stable aneuploids in which p62 has been reported to accumulate [13]. Because activating autophagy by starvation is thought to rescue the autophagy defect in stable aneuploid cells [13], we explain the difference in our results by suggesting that in stable single chromosome aneuploids there may be insufficient activation of autophagy, while our CIN models give robust autophagy induction, giving effective removal of p62 as well as mitophagy. The genomic imbalance caused by gain of a single chromosome in human cells is relatively minor (< 9%) while aneuploidy for even a single major chromosome in *Drosophila* can alter the genome by more than 30%, so it is not surprising that our CIN model evokes more robust cellular adaptive responses.

We found that activation of autophagy was vital for the survival of CIN cells, as it is for stable trisomics [35], as inhibiting autophagy led to increased oxidative stress, DNA damage and massive apoptosis in CIN cells (Figure 2, Figure 3, Supplementary Figure S1 and S2). On the other hand, we found that enhancing autophagic flux by depletion of Tor could significantly reduce the level of reactive oxygen species (ROS) and apoptosis in CIN cells (Figure 4 and Supplementary Figure S3). Depleting Tor has numerous cellular effects including reduced translation, lipid and nucleotide synthesis and increased cap independent translation [36, 37], all of which are likely to impact CIN cell survival. However, the significance of autophagy as part of this response is clear from the cell death when autophagy is specifically reduced: our findings are consistent with a protective role for autophagy in response to aneuploidy and the redox stress that comes with aneuploidy [28, 38]. It is interesting that the CIN should invoke a protective response as well as the cell lethal immune responses that remove defective cells [22]. Our interpretation is that autophagy is a buffering process that can manage stresses within the normal range and prevent any auto-immune responses, but this has a limit beyond which damaged mitochondria accumulate, the redox stress signals are produced and the immune response is triggered.

Autophagy has been reported to suppress CIN in tumour cells, however, the detailed mechanism is not clear [31]. In this study, we found that enhancing autophagic flux could reduce the level of CIN in *Drosophila* proliferating cells (Figure 5). We examined the possibility of chromatid removal by autophagy [39], but failed to observe any co-localization of DNA with autophagosomes in mitotic cells (Figure 5 and data not shown), suggesting that autophagy does not directly degrade lagging chromosomes in our CIN models. However, we found co-localization of mitochondria and autophagosomes suggesting that defective mitochondria are degraded by autophagy (mitophagy) (Figure 5). Furthermore, we found that overexpression of the mitophagy regulator *parkin* [30, 40] could significantly rescue the level of ROS and apoptosis in CIN cells while depletion of Parkin to block mitochondrial turnover had the opposite effect (Supplementary Figure S5). Although mitochondria are built to tolerate ROS by producing localized antioxidants such as superoxide dismutase, it is not surprising that the high levels of ROS produced by mitochondria in CIN cells [21] should damage them to the point where they require mitophagy [41]. In the absence of this quality control system, we observed high rates of DNA damage. Our interpretation of these data is that the CIN rate is responding primarily to the level of DNA damage: when autophagy is increased the level of ROS and DNA damage in CIN cells is lowered, so the CIN rate is correspondingly less. DNA damage is a well-described driver of CIN rates [42] that we have shown is responsive to ROS levels in CIN cells [21], however other responses to autophagy may also contribute. While decreasing autophagy might be an effective mechanism for pre-tumourous tissue to increase its mutation rate, tumours need to balance their level of CIN to avoid intolerable genotoxic stress [43]. Modulating mitophagy is likely to play a key part in fine tuning the rate of CIN to an adaptive level.

**Figure 6:**
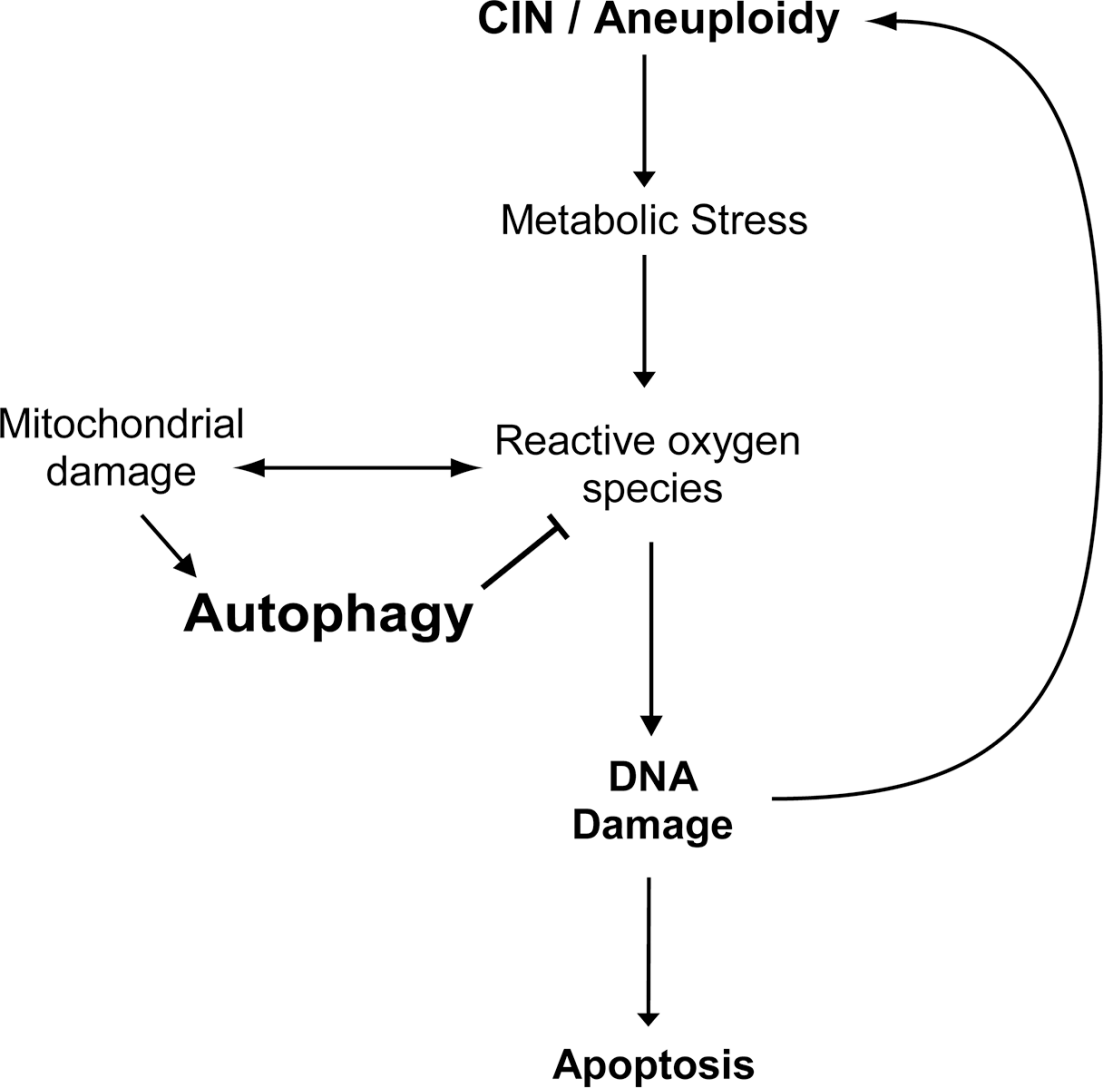
A model for the effect of autophagy on the survival of CIN cells Chromosomal instability leads to metabolic stress and the production of reactive oxygen species, which in turn cause defective mitochondria and further oxidative stress. Autophagy can be activated to effectively remove the defective mitochondria and thus reduce the level of oxidative stress, DNA damage and apoptosis in CIN cells. Moreover, autophagy could reduce the level of CIN by reducing DNA damage in CIN cells.

In conclusion, our data suggests that autophagy effectively removes defective mitochondria in CIN cells thus reducing the level of ROS, DNA damage and apoptosis in CIN cells. Moreover, the reduced level of ROS and DNA damage further mitigate the level of CIN (Figure 6). Our study reveals a mechanism by which autophagy limits CIN in cells, which underscores the importance of understanding autophagy in CIN tumour treatment.

## MATERIALS AND METHODS

### Drosophila stocks

The fly stocks used in this paper are as follows: *mad2*-RNAi (VDRC 47918), *Rad21*-RNAi (Bloomington #36786), *mcherry*-*Atg8a* [24], *Atg1*-RNAi (VDRC 16133), *Atg18*-RNAi (VDRC 22643), *Tor*-RNAi (VDRC 35578), mCherry-RNAi (Bloomington 35785), *Parkin*-RNAi (VDRC 104363), UAS-*park* (Bloomington #34746), *UAS-mito-GFP* (Bloomington #8442), *engrailed-Gal4* (Bloomington #30564).

### Lysotracker and Acridine Orange staining

Lysotracker staining was used to detect autophagy in larval wing imaginal discs. The dissected imaginal discs were transferred from PBS and incubated in 1 μM lysotracker (Lysotracker red DND-99, Invitrogen) and 6 μg/ml Hoechst (Hoechst 33342, Sigma) for 5 mins and then mounted to a slide with PBS for microscopy after a quick wash in PBS.

Acridine Orange (Invitrogen) was used to identify the level of cell death in the *engrailed* driven third instar larval wing discs. Imaginal wing discs were selected and dissected in PBS, then stained for 2 min in a 1 μM Acridine Orange solution, rinsed briefly, mounted and imaged in PBS. For quantitation, the stain was normalized by subtracting the average Acridine orange signal of the wild type anterior compartment from the average Acridine orange signal in the *engrailed*-Gal4 driven mutant posterior compartment (marked with m*CD8-GFP*), using ImageJ software. To reduce noise, background subtraction (rolling ball radius at 10 pixels) was done in all the images [12].

### Oxidative stress assay

The fluorogenic probe CellROX (Life Technologies) was used to measure the level of reactive oxygen species (ROS) in CIN cells as detailed in [21]. Briefly, imaginal wing discs from third instar larvae were dissected in D22 media (pH 6.8), then placed in 5 μM CellROX in D22 media (D22 insect culture medium: pH 6.8) for 15 minutes in the dark at room temperature. Discs were then quickly washed in PBS and fixed in 3.7% formaldehyde for 5 minutes. Fixed discs were then mounted in 80% glycerol and observed under a fluorescence microscope.

### Immunostaining

The standard method for immunostaining in our lab has been used in this study [21]. Briefly, wing discs were dissected in PBS, fixed in 3.7% formaldehyde for 20 min, then blocked with PBS plus 0.2% Tween-20 before being incubated with primary antibodies, usually overnight at 4^°^C. Discs were washed in PBSTw before and after secondary antibodies were added for 2 hrs at room temperature, and discs were mounted in 80% glycerol. All images are of 3^rd^ instar larval wing discs. The region expressing RNAi was marked with CD8-GFP by the use of *UAS-CD8-GFP* driven in the same *engrailed* pattern as the RNAi transcript as indicated in the figure legends. Where shown, RNAi to the non-*Drosophila* gene mCherry was used as a control instead of wild type to ensure that expression of an RNAi construct in this tissue did not have an effect irrespective of its target. The details of antibodies used in this study are listed here: The primary antibodies are Rabbit anti-cleaved caspase3 (D175, 1:100) (Cell Signalling); Rabbit anti-Phospho-H2AVD (Rockland, Lot# 30352, 1:700), rabbit anti-*Drosophila* p62 (generous gift of Prof. Juhasz, Budapest, 1:150). The secondary antibody is CY3 anti-rabbit (1:200). Quantification of cleaved Caspase3 staining was normalized by subtracting the average signal from the wild type anterior compartment from the average signal in the *engrailed*-Gal4 driven mutant posterior compartment (marked with m*CD8-GFP*), using ImageJ software. A minimum of 9 discs were used for each quantitation as described in the relevant figure legends.

### Imaging

The microscopy of CellROX, Acridine Orange staining, and immuno-staining was done on a Zeiss Axioplan2 microscope. The microscopy of mCherry-Atg8 and mitoGFP co-localization was obtained using a Zeiss LSM-700 confocal inverted microscope with Argon ion 488 nm (14 mW) and Green HeNe 543 nm (1.5 mW) lasers. The dual labelled samples were imaged with two separate channels (PMT tubes) in a sequential setting. Green fluorescence was excited with an Ar 488 nm laser line, and the emission was viewed through a HQ515/30 nm narrow band barrier filter in PMT1. Red fluorescence was excited with a HeNe 543 nm laser line, and the emission was viewed through a long pass barrier filter (E570LP) in PMT2. Confocal images shown are from a single plane of focus and show structures that are not visible when imaged 1.5 μm higher or lower, suggesting colocalization rather than overlap of out-of-focus signals. Images were captured using the Zen (Jena, Germany) software and compiled using Photoshop and Illustrator CS5 (Adobe).

### Data analysis

Further details of data analysis are described in [21] and [22], including normalization of the signal from half wing discs to compensate for variations in staining intensity and background subtraction for Acridine Orange staining. Quantitation was carried out using ImageJ and statistical analysis was carried out using GraphPad Prism. All error bars represent 95% confidence intervals for the mean, and measures of the difference in means were done using two-tailed *t* tests with Welsh's correction.

## SUPPLEMENTARY MATERIALS FIGURES


