# Supplementary material for: Autophagy regulates the survival of cells with chromosomal instability
Source: Oncotarget. 2016 Aug 31;7(39):63913–23. doi: 10.18632/oncotarget.11736 (PMC5325413; doi:10.18632/oncotarget.11736)
Supplement: Supplementary file 1 [file oncotarget-07-63913-s001.pdf]

Autophagy regulates the survival of cells with chromosomal instability

Supplementary Materials

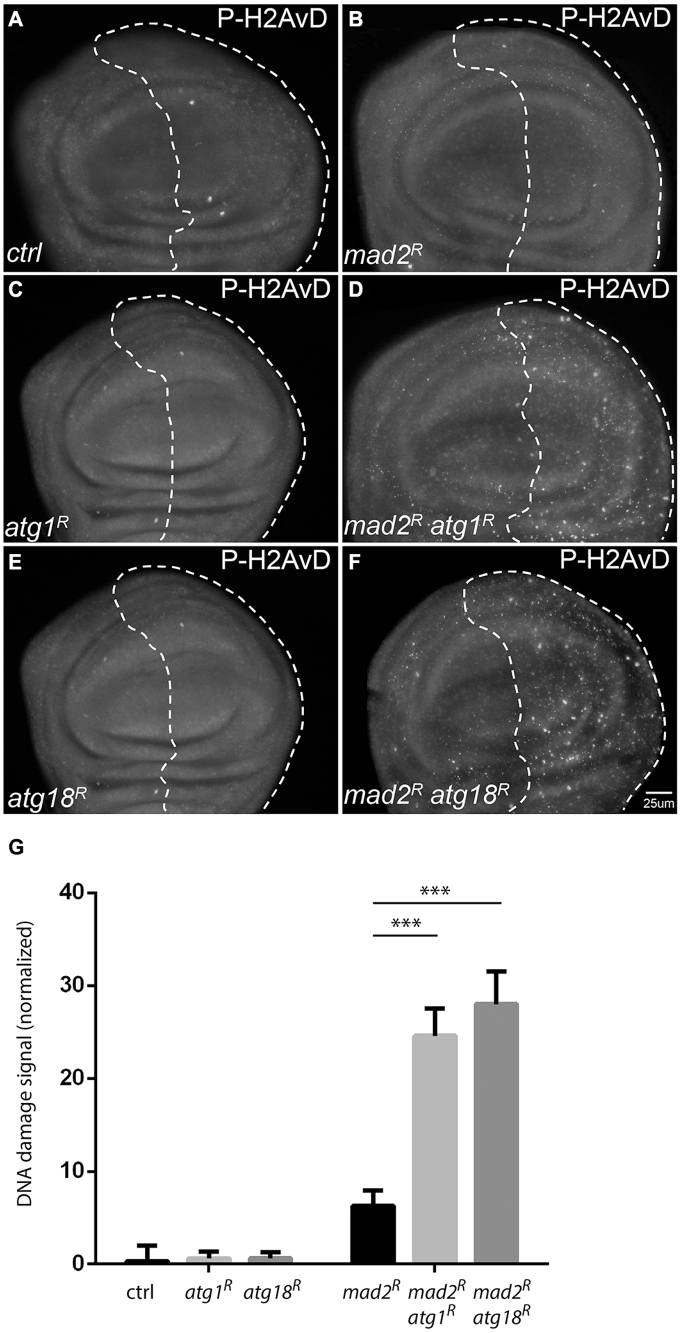

Supplementary Figure S1: The effect of knocking down autophagy on the level of DNA damage in CIN cells. Anti-phosphorylated H2AvD antibody was used to detect the level of DNA damage. Knocking down either Atg1 (**C**, *engrailed > Gal4*, *UAS-CD8-GFP,* UAS-*Atg1*^{RNAi}) or Atg18 (**E**, *engrailed > Gal4*, *UAS-CD8-GFP,* UAS-*Atg18*^{RNAi}) did not cause DNA damage in proliferating cells. However, knocking down Atg1 (**D**) or Atg18 (**F**) significantly increased the level of DNA damage in CIN cells compared to the CIN alone control (**B**). Quantitation of the number of P-H2AvD puncta is shown in (**G**). For all genotypes $n \geq 9$ and the error bars show 95% confidence intervals around the mean. The p values were calculated using two-tailed t tests with Welch's correction, and were < 0.001 for both marked comparisons.

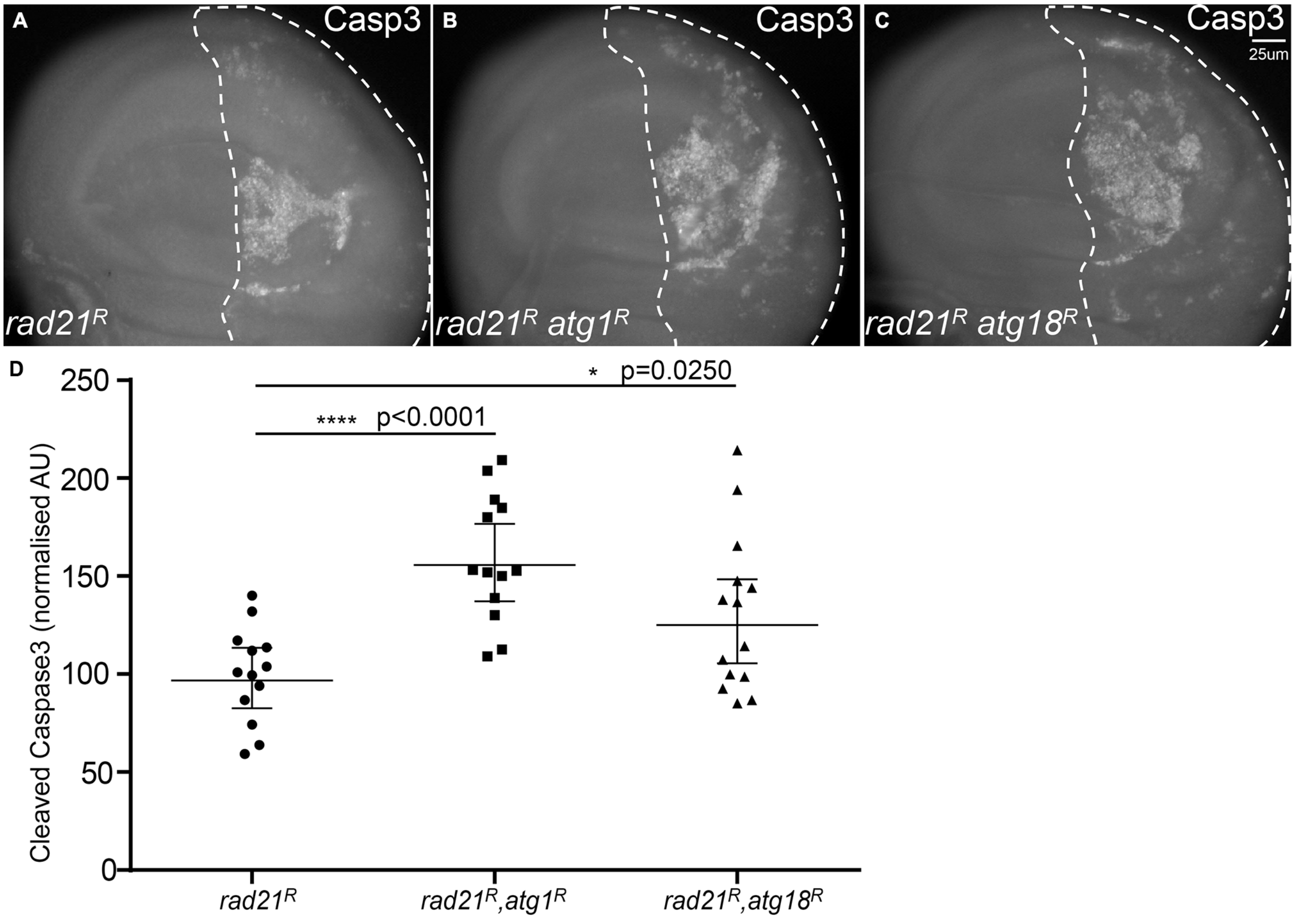

Supplementary Figure S2: The effect of knocking down autophagy on cell death in CIN cells. Anti-cleaved caspase3 antibody was used to detect the level of apoptosis. Knocking down either Atg1 ((**B**) *engrailed > Gal4*, *UAS-CD8-GFP, UAS-rad21^{RNAi} UAS-Dicer2*, UAS-*atg1^{RNAi}*) or Atg18 ((**C**) *engrailed > Gal4*, *UAS-CD8-GFP, UAS-rad21^{RNAi} UAS-Dicer2,* UAS-*atg18RNAi*) significantly increased the level of apoptosis in CIN cells induced by Rad21 depletion compared to the CIN alone control ((**A**) *engrailed > Gal4*, *UAS-CD8-GFP, UAS-rad21^{RNAi} UAS-Dicer2*). Quantification of the cleaved caspase3 staining is shown in (**D**). For all genotypes $n > 12$ and the error bars show 95% confidence intervals around the mean. The p values were calculated using two-tailed t tests with Welch's correction.

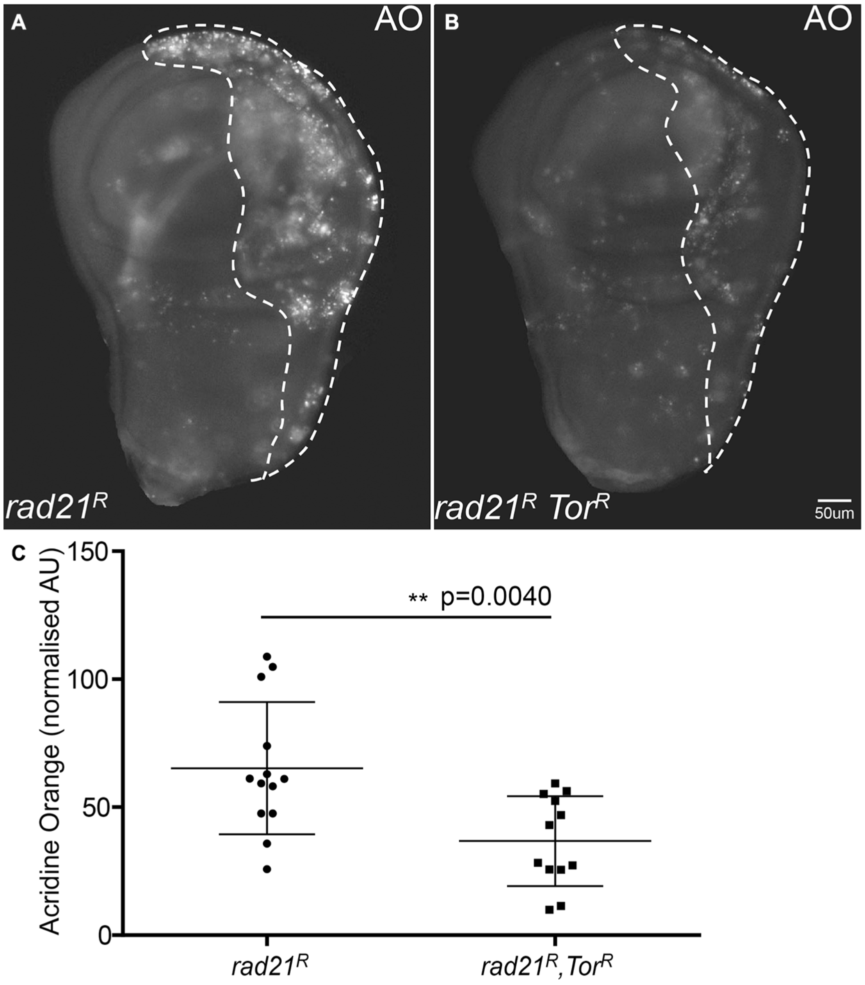

Supplementary Figure S3: The effect of enhancing autophagy on the cell death in CIN cells. Acridine orange staining was used to detect the level of cell death. Enhancing autophagy by Tor knock down ((**B**) *engrailed* > *Gal4*, *UAS-CD8-GFP,* UAS-*Tor^{RNAi}*, UAS-*rad21^{RNAi}*, UAS-*Dicer2*) significantly reduced the level of cell death in CIN cells compared to the CIN alone control ((**A**) *engrailed* > *Gal4*, *UAS-CD8-GFP,* UAS-*rad21^{RNAi}*, UAS-*Dicer2*). Quantification of the Acridine Orange staining is shown in (**C**). In both cases $n \geq 12$ and the error bars show 95% confidence intervals around the mean. The *p* values were calculated using two-tailed *t*-tests with Welch's correction.

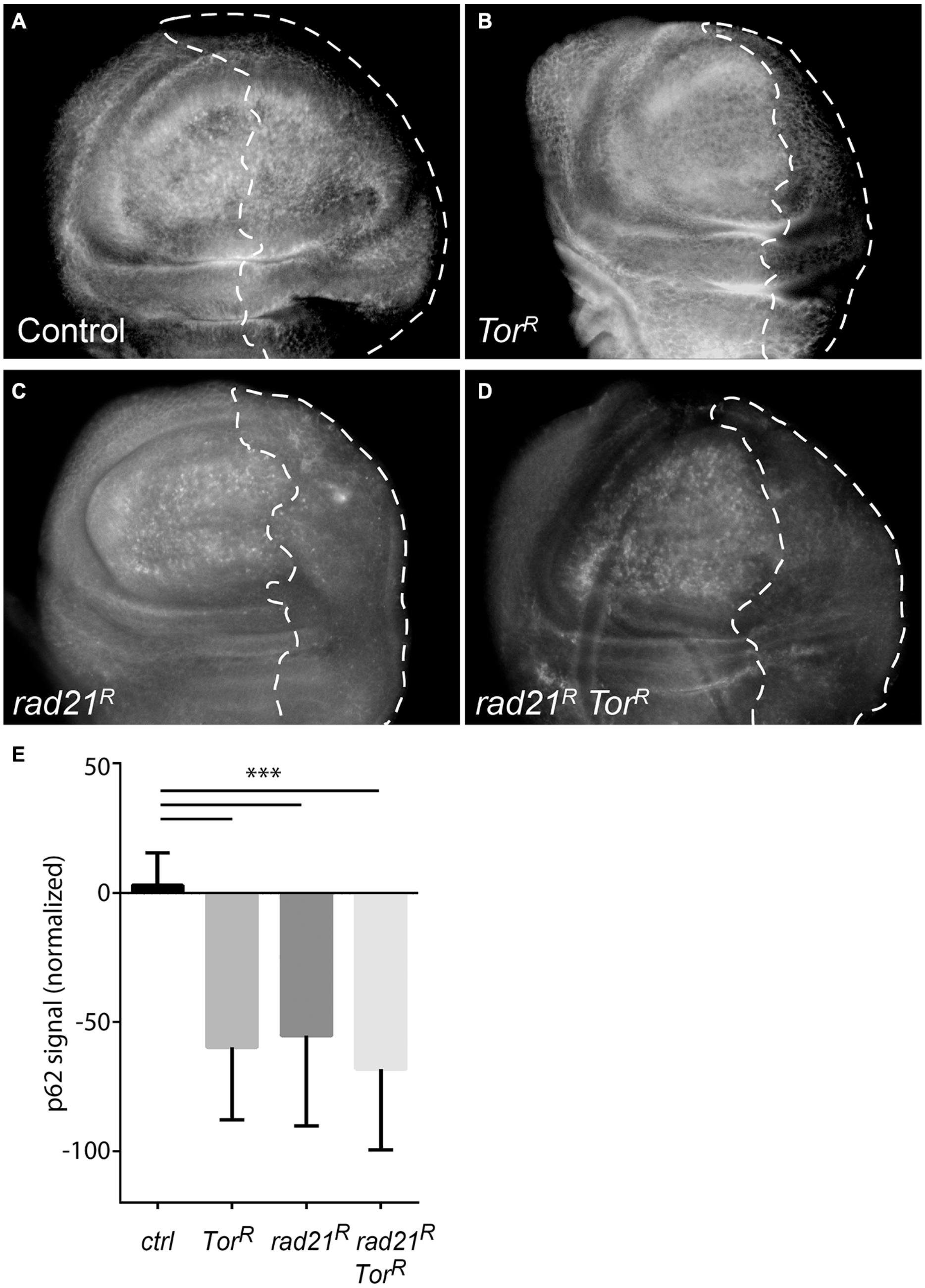

Supplementary Figure S4: Knockdown of Tor gives increased autophagy as measured by p62 staining. The level of p62, an autophagy substrate, was measured to test the level of autophagic flux in third instar larval wing discs. When Tor was depleted by RNAi in the posterior half of the disc ((**B**) *engrailed* > *Gal4*, *UAS-CD8-GFP,* UAS-*Tor*^{RNAi}, dotted lines), less p62 staining was observed compared to control discs ((**A**) *engrailed* > *Gal4*, *UAS-CD8-GFP, UAS-mCherry*^{RNAi}). Depletion of Rad21 (**C**) also reduced p62 levels, as did depletion of Tor and Rad21 (**D**). Quantification of the p62 levels is shown in (**E**). In all cases $n > 16$ and p values were calculated using two-tailed t-tests with Welch's correction, giving $p < 0.001$ for all marked comparisons.

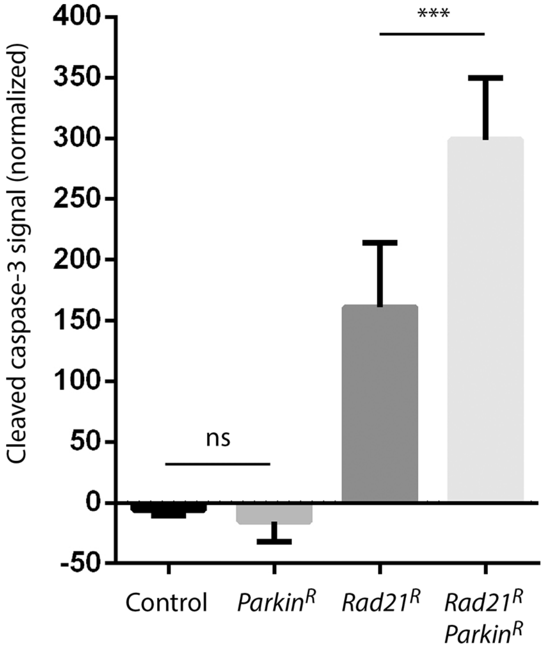

Supplementary Figure S5: Decreased mitophagy increases apoptosis of CIN cells but not normal cells. Staining for cleaved caspase3 was used to measure the rate of apoptosis in CIN cells compared to normal cells in third instar larval wing discs. In control discs (*engrailed > Gal4*, *UAS-CD8-GFP,* UAS-*mCherry*^{RNAi}) or when Parkin was depleted to decrease mitophagy (*engrailed > Gal4*, *UAS-CD8-GFP,* UAS-*Parkin*^{RNAi}), apoptosis was not induced in these proliferating cells. Apoptosis was induced by depleting Rad21 (*engrailed > Gal4, UAS-Rad21*^{RNAi}, UAS-*Dicer2,* UAS-*mCherry*^{RNAi}), and this was significantly increased when Parkin was also depleted in CIN cells (*engrailed > Gal4, UAS-Rad21*^{RNAi}, UAS-*Dicer2,* UAS-*Parkin*^{RNAi}). In all cases $n \geq 9$ and the error bars show 95% confidence intervals around the mean. The p values were calculated using two-tailed t-tests with Welch's correction, the indicated significant variation in means gave $p < 0.0001$.
